# A successful pregnancy and parturition in a patient with anuria undergoing maintenance hemodialysis for 6 years: a case report of a 3-year-follow-up

**DOI:** 10.1186/s12884-015-0642-9

**Published:** 2015-09-14

**Authors:** Panxi Yu, Wenqi Diao, Qionglan Tang, Xuefeng Jiang

**Affiliations:** Plastic Surgery Hospital, Chinese Academy of Medical Sciences and Peking Union Medical College, Beijing, China; Department of Clinical Medicine, International School, Ji-nan University, Guangzhou, China; Department of Respiratory Medicine, Peking University Third Hospital, Beijing, China; Department of Pathology, Sun Yat-sen Memorial Hospital of Sun Yat-sen University, Guangzhou, China; Department of Obstetrics and Gynecology, the First Affiliated Hospital of Ji-nan University, Guangzhou, China

**Keywords:** Pregnancy, Hemodialysis, Chronic renal failure

## Abstract

**Background:**

Pregnancies in hemodialysis patients are uncommon and difficult to study. Although the chance of a successful pregnancy and parturition in hemodialysis women has increased over the years, it still remains extremely low with a high maternal and fetal mortality and morbidity rate.

**Case presentation:**

We reported a case of successful pregnancy and parturition in a 22-year-old Chinese female in uremic stage of chronic renal failure and undergoing maintenance hemodialysis (three sessions a week) for 6 years. At the 22nd gestational week, she was diagnosed as pregnant by ultrasound, and started an enhanced hemodialysis routine (Five sessions a week). At the 32nd gestational week, she got hospitalized and received hemodialysis more frequently (seven sessions a week). Based on the initial diagnoses, including uremic stage of chronic renal failure, stage-3 hypertension, single pregnancy of 32nd gestational week, single umbilical artery and polyhydramnios, a drug therapy consisting of compound amino acid, fructosediphosphate sodium, 10 % L-carnitine, erythropoietin, polyferose, amlodipine, isosorbidedinitrate, low-molecular weight-heparin, multivitamins and folic acid was given, and daily examination of the mother and fetus was performed. Under the joint efforts of various departments, the patient underwent caesarean section at the 34th gestational week due to progressive uterine contraction and gave birth to a female, well-being baby weighing 1470 g. It has been more than 3 years since the parturition. The mother has returned to the previous hemodialysis routine, and the child has been growing up healthily.

**Conclusion:**

Although pregnancy in hemodialysis patients is rare, with a high rate of risks. Patients could still gain a good outcome, if we intensify hemodialysis and enhance the collaboration between the patient, nephrologists, obstetricians, neonatologist, nutritionists, and other departments.

## Background

Pregnancies in hemodialysis (HD) patients are rare [1], although the incidence of these pregnancies has increased since 1971 Confortini reported the first successful case [2]. And compared to normal population, there is a very high maternal and fetal mortality and morbidity rate in pregnant women undergoing HD [3, 4]. In order to achieve a successful birth, this situation requires the joint efforts of the patient, nephrologists, obstetricians, neonatologists, nutritionists and other departments [3]. Here we reported a case of successful pregnancy and parturition in a HD patient who was in uremic stage of chronic renal failure (CRF) and reviewed the associated literatures.

## Case presentation

### General information

**Fig. 1:**
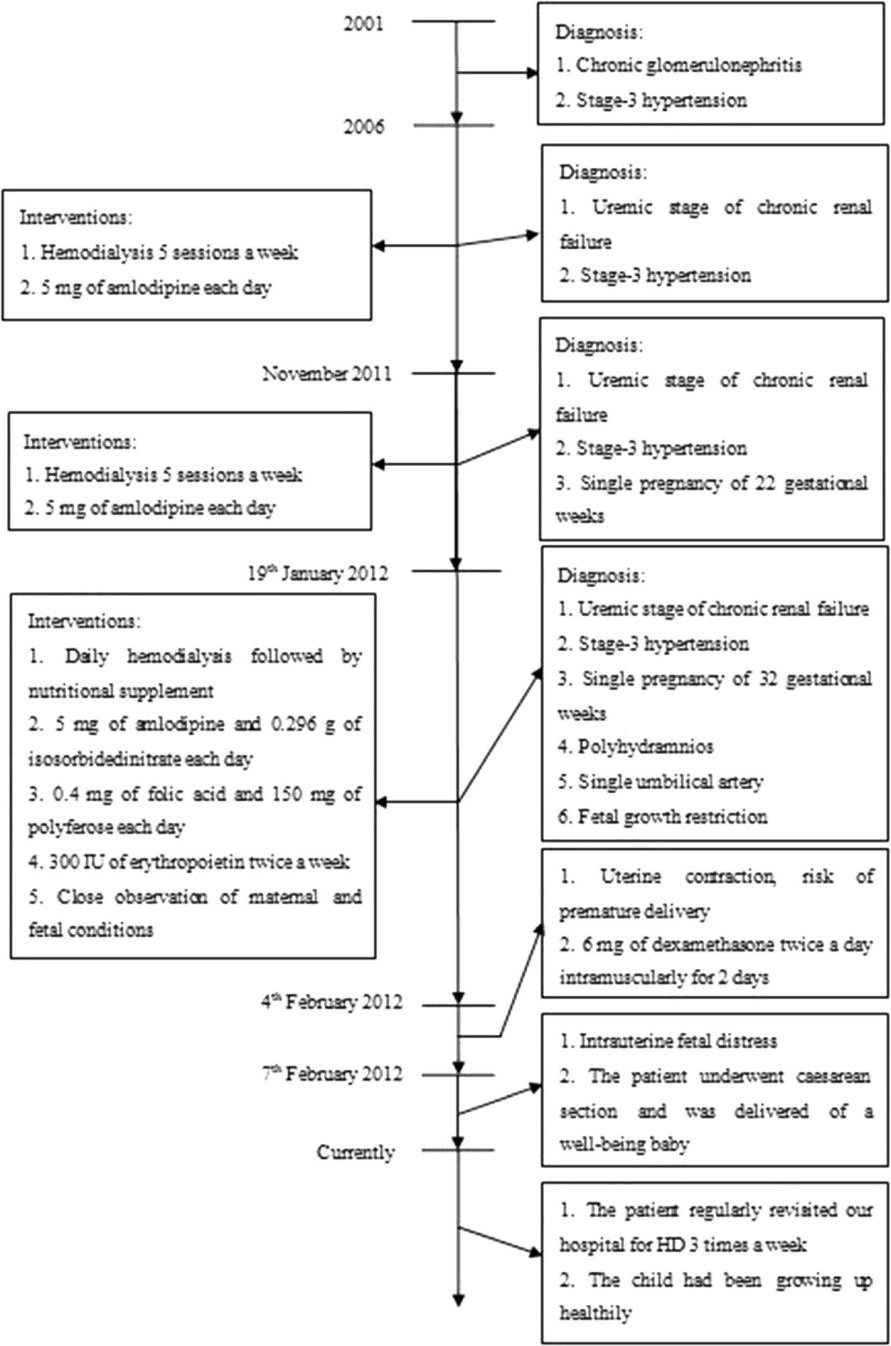
Time scale of the patient care

The patient was a Chinese woman, 22 years old, married and nulliparous. The time scale of the patient care was shown in (Fig. 1).

### Past medical history

At the age of 11 (Year 2001), the patient was diagnosed with chronic glomerulonephritis and stage-3 hypertension without a definite etiology. She irregularly took Chinese herbs (details were unknown) hoping to alleviate the progression of the diseases. Probably due to the treatment “nonfeasance”, at her age of 16, she stepped into the uremic stage of CRF. And in order to ease the disease progression, she started to take 5 mg of amlodipine each day and initiated a routine HD three sessions a week. Since November 2011, the patient started to noticea progressive distending pain of the abdomen, and was found pregnant for 22 gestational weeks (GWs) with polyhydramnios (Amniotic Fluid Index-AFI, was unknown) by ultrasound. From that on, her HD routine was enhanced into five sessions a week.

### Admission condition

On 19th January 2012, the patient came to our hospital. A comprehensive examination was performed. Her blood pressure was 152/86 mmHg, body weight 56.6 kg, height 146.7 cm, and a calculated body mass index was 26.3 kg/m^2^. Her blood tests were: Blood Urea Nitrogen (BUN)13.02 mmol/L, Serum Creatinine (Cr) 422 μg/L, Hemoglobin (Hb) 94 g/L, Hematocrit (Hct) 27 %, Serum Ferritin (SF) 10.8 μg/L, Red Blood Cell (RBC) 3.35 × 10^12^/L, Platelet (PLT) 186 × 10^9^/L, D-Dimer (DD) 0.43 mg/L FEU, Total Protein (TP) 58.8 g/L, Albumin (ALB) 32.6 g/L, Serum Potassium (K) 3.7 mmol/L, Serum Sodium (Na) 137 mmol/L, Serum Chlorine (Cl) 103 mmol/L, Serum Calcium (Ca) 2.08 mmol/L, Creatine Kinase (CK) 95 U/L, Creatine Kinase-MB isoenzyme (CK-MB) 0.6 μg/L, Cardiac Troponin I (cTnI) 0.004 μg/L, and N-Terminal pro-Brain Natriuretic Peptide (NT-proBNP) 124 pg/ml. Abdominal ultrasound revealed: single pregnancy of 32 GWs, single umbilical artery, polyhydramnios (AFI 19.5) and fetal growth restriction (the fetus was as large as that of 29GWs).

### Initial treatment protocol

According to the examination results and past medical history, the patient was initially diagnosed with: CRF (uremic stage), stage-3 hypertension, single pregnancy of 32GWs, single umbilical artery, polyhydramnios, fetal growth restriction, and slight anemia. In order to afford a comprehensive treatment, nephrologists, obstetricians, neonatologists, nutritionists and cardiovascular department together established the following therapeutic regimen:

Daily HD of 240 min was performed on the patient, followed by intravenous supplement of 250 ml of compound amino acid, 20 ml of 10 % L-carnitine, and 10 g of fructose diphosphate sodium. 5 mg of amlodipine and 0.296 g of isosorbidedinitrate were given per day to control the blood pressure. 0.4 mg of folic acid and 150 mg of polyferose each day and 300 IU of erythropoietin (EPO) twice a week were applied to alleviate anemia. Additionally, close observation of maternal and fetal conditions was required: auscultation of fetal heart rate (FHR), calculation of fetal movement, maternal electrocardiogram (ECG), check of maternal vital signs, measurement of maternal abdominal circumference, and maternal blood tests were strictly performed each day. Color Doppler ultrasound was performed once a week. Non-Stress-Test (NST) was performed 3 times a week. Additionally, blood examination of myocardial enzymes was executed in case of heart failure.

### Disease progression

From 4th February 2012, the patient started feeling abdominal intending pain with paroxysmal severe abdominal cramps. Abdominal ultrasound was performed, indicating: single pregnancy of 34^+1^ GWs, single umbilical artery, fetal growth restriction, and polyhydramnios (AFI 22.9). The blood tests were: BUN 14.19 mmol/L, Cr368 μg/L, Hb 90 g/L, Hct 24 %, SF 11.3 μg/L, RBC 3.26 × 10^12^/L, PLT 194 × 10^9^/L, DD 0.24 mg/L FEU, TP 56.8 g/L, ALB 32.3 g/L, K 3.5 mmol/L, Na 139 mmol/L, Cl 108 mmol/L, Ca 2.20 mmol/L, CK 75 U/L, CK-MB 0.3 μg/L, cTnI 0.000 μg/L, NT-proBNP 119 pg/ml. NST confirmed that the paroxysmal abdominal pain was due to uterine contraction, indicating a great risk of premature birth. To facilitate the maturation of fetal lungs, 6 mg of dexamethasone twice a day was given to the patient intramuscularly for 2 days.

**Fig. 2:**
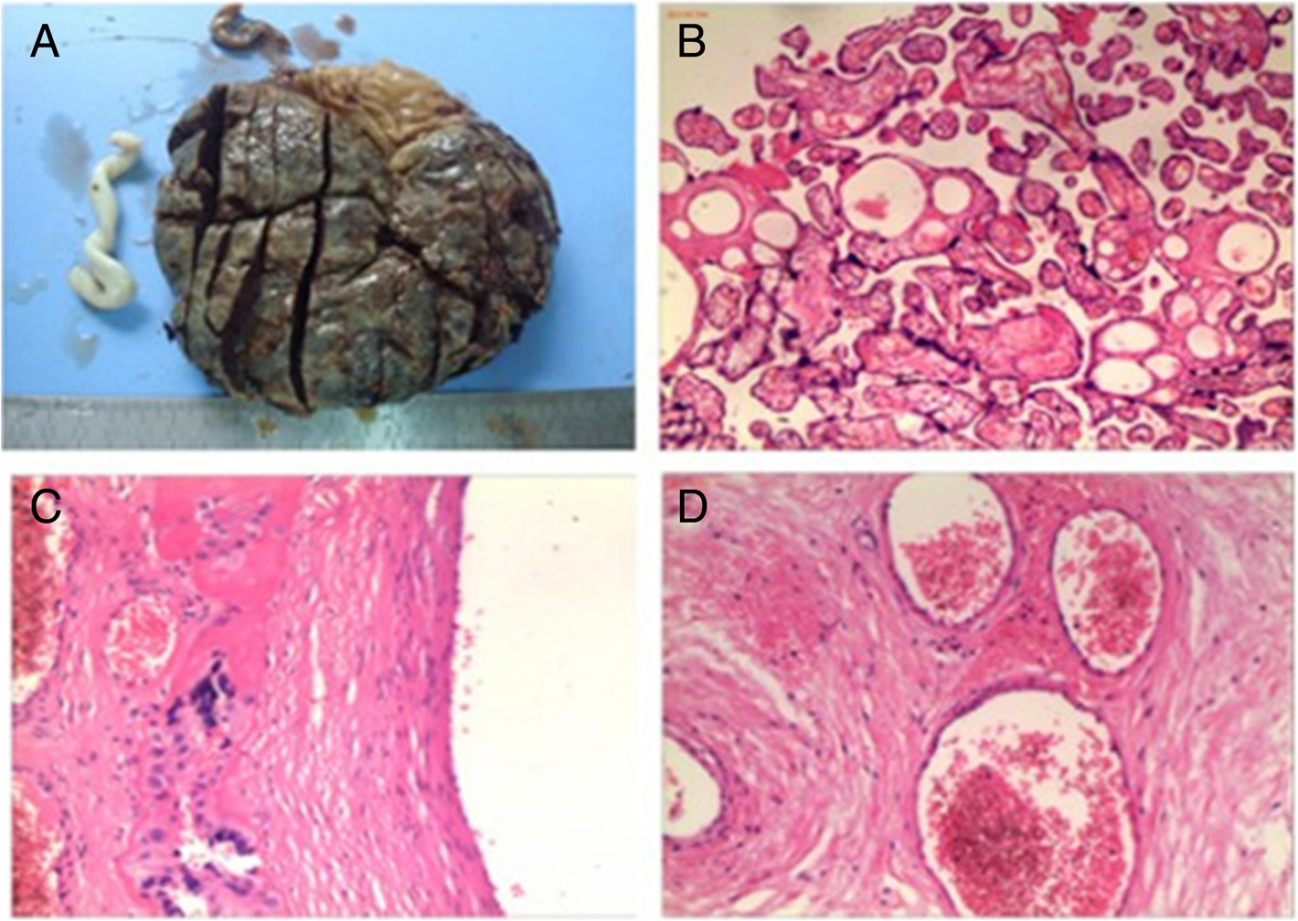
Placenta and its pathological examination. **a** Placenta by naked eyes. The volume is 18 × 13 × (1.5–2) cm, with sections of dull-red color and complete placental membrane, no cyst can be found. **b** Chorionic villi of placenta under microscope (HE × 200). Mild edema and increased syncytial cell nodules can be seen in partial villi, indicating placental dysfunction. **c** A chorionic blood vessel under microscope (HE × 400). Chorionic blood vessel presents hyaline degeneration and fibrous necrosis, according with the diagnosis of hypertension. **d** Umbilical artery under microscope (HE × 400). There is only one artery in umbilical cord, with a thickened wall and a narrow lumen, according with the diagnosis of single umbilical artery

On 7th February 2012 (at the 34^+4th^GW), no fetal movement was observed, and a non-reaction type of NST was presented. The baseline of FHR was 145 beats per minute, without obvious FHR fluctuation even during HD. Intrauterine fetal distress was highly suspected, and so we decided to cease the pregnancy immediately. On that day, the patient underwent caesarean section and was delivered of a female, well-being baby weighing 1470 g. The Apgar score was 5, 8 and 9 at the 1st, 5th and 10th minutes respectively. The placenta was pathologically examined (Fig. 2), and the result revealed placental dysfunction, single umbilical artery and vascular pathological change caused by hypertension.

On 8th February 2012, the patient felt chest tightness and coughed up foamy sputum. In case of heart failure, ECG was performed and blood tests were examined: BUN 13.15 mmol/L, Cr376 μg/L, K 6.6 mmol/L, Na 134 mmol/L, Cl 110 mmol/L, Ca 2.14 mmol/L, CK 158 U/L, CK-MB 3.3 μg/L, cTnI 0.051 μg/L, NT-proBNP 122 pg/ml. Although the ECG and myocardial enzymes did not indicate any abnormity, the transient hyperkalemia could be malicious and fatal to postpartum women with CRF. Therefore HD was imminently performed to alleviate the hyperkalemia. And fortunately, the situation was controlled in time.

### Outcomes and 3-year follow-up

During the following days, the mother was in a stable condition, and there was no evidence of neonatal pathology. Therefore, they were discharged from the hospital on the 8th post-delivery day. The patient still suffered from anuria, and regularly revisited our hospital for HD three times a week.

Recently the mother and child returned to our hospital. The mother’s condition had been stabilized by regular hemolysis, and the child had been growing up healthily.

### Literature review and discussion

Uremia-associated hypothalamo-pituitary dysfunction and malnutrition usually result in amenorrhea and anovulation, and thus disturbances in menstruation and fertility are commonly encountered in women with CRF, leading to a low pregnancy rate in these patients [1, 5]. HD is performed on CRF patients, especially those suffered from anuria, to alleviate the uremic condition. However, disturbance of body homeostasis is a common complication of HD [1, 4, 5]. Long-term nausea and vomiting caused by acid-base and electrolytes imbalance may cover the early pregnancy reactions in HD patients, delay the treatment, and lower the successful rate of HD parturition. Since 1971 Confortini et al. [2] reported the first successful case of HD pregnancy, further cases followed. In 1980 the European Dialysis and Transplant Association (EDTA) reported a pregnancy incidence of 0.9 % [6], while recent publications reported pregnancy in 1.0–7.0 % women on chronic dialysis, and 30–50 % of those pregnancies resulted in delivery of a surviving infant [7]. Although the successful incidence tends to increase, it is still uncommon, and with high maternal and fetal mortality and morbidity rate [3, 4].

In women of childbearing age on dialysis, menstrual irregularity, amenorrhea, sexual dysfunction, nausea and elevated beta-subunit of human chorionic gonadotropin (β-HCG) have been observed in some patients with renal failure which may give a false-positive pregnant test [8, 9]. Early diagnosis is advantageous for maternal fitness and fetal viability, since it allows intensified dialysis early in pregnancy and an early review of medications contraindicated during pregnancy. In our case, the patient became pregnant after 6 years of HD, and she was not found pregnant until the 22nd GW. The late diagnosis might have delayed the intensive antenatal care and reduced the successful chance. However, the adequate HD might largely contribute to the successful maintenance of pregnancy and parturition in our case [1, 4, 5].

Polyhydramnios is a common complication in pregnant women undergoing HD with an incidence ranging from 30–70 % [8–10]. It has been reported that intensified HD could alleviate polyhydramnios by reducing fluid over-burden and lowering osmotic pressure [7, 11]. In our case, the patient received 5 HD sessions a week since the 22nd GW. Her AFI was 19.5 at the 32nd GW, and started daily HD. It seemed that the intensified HD didn’t reduce the amniotic fluidto a better level, as AFI increased to 22.9 at the 34th^+1^ GW, yet the pregnant outcome and fetal viability was impressive. This result is corresponding to the previous finding that polyhydramnios was associated with a favorable fetal outcome in pregnant women undergoing long-term HD [12, 13].

The pathophysiology of polyhydramnios has not yet been clarified. An influence of the fetal osmotic diuresis, caused by the increased concentration of urea and related substances in maternal blood, has been proposed [12]. Although intensified HD may ameliorate the exacerbation of polyhydramnios, the influence of osmotic diuresis may overwhelm. This may explain why the AFI failed to decrease despite of daily HD. Additionally, fetuses with uteroplacental insufficiency habitually shunt blood away from nonessential organs, such as kidneys, to decrease urine output and thus amniotic fluid volume, leading to oligohydramnios [4]. The presence of polyhrydramnios may be an indirect evidence of adequate placental function and therefore be associated with better fetal prognosis. Further studies are in need to discuss the relationship between the presence of polyhydramnios and the “doses” of HD.

Hypertension is the most frequently reported maternal complication in this population [14]. Due to combination of hypertention and inefficient renal function, the incidence of pre-eclampsia is alarmingly high, occurring in 50 % of pregnant women with pre-existing renal diseases [15, 16]. The presence of pre-eclampsia had a negative impact on the successful delivery rate and accounted for 57 % of perinatal deaths, and interventions lowering the incidence or severity of pre-eclampsia may potentially improve fetal outcome [4]. However, the fluctuant blood pressure, proteinuria as well as BUN and Cr level make the diagnosis of pre-eclampsia in patients undergoing HD exceedingly difficult, delaying the early medical intervention and increasing the maternal and fetal morbidity and mortality.

In this case, the patient had taken amlodipine to control blood pressure for 6 years. When being found pregnant, she continued amlodipine therapy and started intensified HD. In case of pre-eclampsia, blood pressure, blood routine test, coagulation, and heart function was frequently measured, and Color Doppler ultrasound involving the uterine arteries was performed at least once a week. This patient’s renal dysfunction and hypertion-before-pregnancy awfully increased the risk of pre-eclampsia. Yet fortunately there were neither clinical manifestations of pre-eclampsia such as severe headache, blurred vision, and palpitation, nor obvious abnormality of the aforementioned tests. The combination of antihypertensive medication and enhanced HD might facilitate the balance of body fluid and stabilization of blood pressure, contributing to the successful outcome.

Low dose of aspirin had been applied to prevent pregnancy-induced hypertension safely [17, 18]. However, animal studies have shown the increased risk of congenital anomalies caused by aspirin, and data from human studies remains conflicting [19]. Moreover, the possible preventive effect of aspirin aims at the early pregnancy. The patient came to our hospital at the 32nd GW, and with a considerable risk of premature delivery. Therefore, aspirin was not applied to this patient.

Anemia occurs during pregnancy presumably due to the hemodilution caused by greater vascular volume, EPO resistance as a result of increased cytokine production during pregnancy, and high demand of red blood cell production for fetal growth [14]. Pregnant women undergoing HD are more likely to have anemia, because of the exceeding loss of iron and red blood cells from frequent HD. Previous studies have shown an increased rate of preterm delivery in women with anemia [20, 21]. The widely accepted indicators of anemia, hemoglobin level and hematocrit, have also been found positively related to the successful rate of parturition and fetal birth weight [22]. Therefore, EPO are regularly given to pregnant dialysis patients, with doses requiring an increase of about 50 %. Also, frequent monitoring of hemoglobin level and hematocrit is required, with a target value of 100–110 g/L and >30 % respectively [4, 23]. In our case, 0.4 mg folic acid and 150 mg of polyferoseeach day and 300 IU of erythropoietin twice a week were given to the patient. Her hemoglobin level and hematocrit were kept stable and near the target value.

Maternal dry weight and weight gain should be regularly evaluated and adjustedaccording to the estimated weight of the placenta and fetus as well as the increase in plasma volume. In the first trimester, the mother should gain a minimum of 1–1.5 kg per week. After this, weight should increase by 0.45–1 kg per week [14]. There is little information on the nutritional status of pregnant dialysis patients, but 1 g/kg/day protein intake for the mother plus 20 g/day for fetal development has been suggested [6, 14, 23].

In maintenance HD, carnitine is lost through dialytic membranes, leading in selected patients to carnitine depletion with a relative increase of the esterified forms [24]. The concentration of plasma amino acids and glucose also falls after HD, resulting a state of fasting and wasting [25]. Thus the intravenous supplement of compound amino acid, L-carnitine, and fructose diphosphate sodium were given to improve the nutritional status of the patient after each HD session.

Fetal anomalies, including spina bifida, encephalocele, cystic hygroma, valvular incompetence, and kyphoscoliosis, belong to the worst situations of pregnancy. The risk factors and/or indications of fetal anomalies include poly- and oligohydramnios, single umbilical artery, maternal diseases such as diabetes, hypertension and infections [26]. In our case, the patient was diagnosed with polyhydramnios, single umbilical artery, anemia, hypertension, and uremic stage of CRF. Therefore the incidence of fetal anomaly is exceedingly high. We carried on color Doppler ultrasound for the patient once a week tomonitor the fetal condition, and fortunately this was a well-being fetus.

Maternal mortality is relatively low and less reported [1, 4]. Cesarean section delivery is common among women on dialysis and is most often prompted by premature rupture of membranes. Infants born to women on HD are usually premature, with multiple causes, such as polyhydramnios, maternal hypertension, premature rupture of the membranes and et al. In our case, the patient underwent progressive uterine contraction at the 34th GW, and intrauterine fetal distress was indicated by NST. Thus we ceased the pregnancy by caesarean section and delivered a female, well-being baby of which the weight was 1470 g. The patient recovered satisfactorily and returned to HD regimen of 3 sessions a week with good tolerance. Currently, the baby hasn’t yet had any obvious pathological change.

## Conclusions

The pregnancy of women on chronic dialysis is at very high risk but it should reach a good outcome with multidisciplinary management, by nephrologists, obstetricians, neonatologists, nutritionists and other departments. We advise that all aspects of HD, including duration, adequacy, nutrition, anemia, and blood pressure control should be closely followed throughout the course of pregnancy. Finally, since pregnancy can occur in women on dialysis, health care providers should discuss fertility and contraception with their premenopausal dialysis patients.

## Consent

Written informed consent was obtained from the patient for publication of this Case report and accompanying images. A copy of the written consent is available for review by the Editor of this journal.

## Abbreviations

HD: Hemodialysis
CRF: Chronic renal failure
BUN: Blood urea nitrogen
Cr: Serum creatinine
Hb: Hemoglobin
Hct: Hematocrit
SF: Serum ferritin
RBC: Red blood cell
PLT: Platelet
DD: D-Dimer
TP: Total protein
ALB: Albumin
K: Serum potassium
Na: Serum sodium
Cl: Serum chlorine
Ca: Serum calcium
CK: Creatine kinase
CK-MB: Creatine kinase-MB isoenzyme
cTnI: Cardiac troponin I
NT-proBNP: N-terminal pro-brain natriuretic peptide
EPO: Erythropoietin
FHR: Fetal heart rate
ECG: Electrocardiogram
NST: Non-stress-test

## References

[1] Hou S (2010). Pregnancy in women treated with dialysis: lessons from a large series over 20 years. Am J Kidney Dis.

[2] Confortini PGG, Ancona G (1971). Full term pregnancy and successful delivery in a patient on chronic hemodialysis. ProcEur Dial Transplant Assoc.

[3] Reddy SS, Holley JL (2007). Management of the pregnant chronic dialysis patient. Adv Chronic Kidney Dis.

[4] Luders C, Castro MC, Titan SM, De Castro I, Elias RM, Abensur H, Romao JE (2010). Obstetric outcome in pregnant women on long-term dialysis: a case series. Am J Kidney Dis.

[5] Kazancioglu R, Sahin S, Has R, Turkmen A, Ergin-Karadayi H, Ibrahimoglu L, Bozfakioglu S (2003). The outcome of pregnancy among patients receiving hemodialysis treatment. Clin Nephrol.

[6] Successful pregnancies in women treated by dialysis and kidney transplantation. Report from the Registration Committee of the European Dialysis and Transplant Association. Br J Obstet Gynaecol 1980, 87(10):839–845. 10.1111/j.1471-0528.1980.tb04434.x 7000160

[7] Holley JL, Reddy SS (2003). Pregnancy in dialysis patients: a review of outcomes, complications, and management. Semin Dial.

[8] Anantharaman P, Schmidt RJ (2007). Sexual function in chronic kidney disease. Adv Chronic Kidney Dis.

[9] Palmer BF (2003). Sexual dysfunction in men and women with chronic kidney disease and end-stage kidney disease. Adv Ren Replace Ther.

[10] Chou CY, Ting IW, Lin TH, Lee CN (2008). Pregnancy in patients on chronic dialysis: a single center experience and combined analysis of reported results. Eur J Obstet Gynecol Reprod Biol.

[11] Moranne O, Samouelian V, Lapeyre F, Pagniez D, Subtil D, Dequiedt P, Boulanger E (2004). [Pregnancy and hemodialysis]. Nephrologie.

[12] Malik GH, Al-Harbi A, Al-Mohaya S, Dohaimi H, Kechrid M, Shetaia MS, Al-Hassan AO, Quiapos LS (2005). Pregnancy in patients on dialysis--experience at a referral center. J Assoc Physicians India.

[13] Hou SH (1994). Frequency and outcome of pregnancy in women on dialysis. Am J Kidney Dis.

[14] Giatras I, Levy DP, Malone FD, Carlson JA, Jungers P (1998). Pregnancy during dialysis: case report and management guidelines. Nephrol Dial Transplant.

[15] Fischer MJ (2007). Chronic kidney disease and pregnancy: maternal and fetal outcomes. Advances in chronic kidney disease.

[16] Vidaeff AC, Yeomans ER, Ramin SM (2008). Pregnancy in women with renal disease. Part I: general principles. Am J Perinatol.

[17] Duley L, Henderson-Smart DJ, Meher S, King JF (2007). Antiplatelet agents for preventing pre-eclampsia and its complications. Cochrane Database Syst Rev.

[18] Coomarasamy A, Honest H, Papaioannou S, Gee H, Khan KS (2003). Aspirin for prevention of preeclampsia in women with historical risk factors: a systematic review. Obstet Gynecol.

[19] Kozer E, Nikfar S, Costei A, Boskovic R, Nulman I, Koren G (2002). Aspirin consumption during the first trimester of pregnancy and congenital anomalies: a meta-analysis. Am J Obstet Gynecol.

[20] Scanlon KS, Yip R, Schieve LA, Cogswell ME (2000). High and low hemoglobin levels during pregnancy: differential risks for preterm birth and small for gestational age. Obstet Gynecol.

[21] Xiong X, Buekens P, Alexander S, Demianczuk N, Wollast E (2000). Anemia during pregnancy and birth outcome: a meta-analysis. Am J Perinatol.

[22] Asamiya Y, Otsubo S, Matsuda Y, Kimata N, Kikuchi K, Miwa N, Uchida K, Mineshima M, Mitani M, Ohta H (2009). The importance of low blood urea nitrogen levels in pregnant patients undergoing hemodialysis to optimize birth weight and gestational age. Kidney Int.

[23] Hull AR (1998). More dialysis appears beneficial for pregnant ESRD patients (at least in Belgium). Am J Kidney Dis.

[24] Guarnieri G, Situlin R, Biolo G (2001). Carnitine metabolism in uremia. Am J Kidney Dis.

[25] Navarro JF, Mora C, Leon C, Martin-Del Rio R, Macia ML, Gallego E, Chahin J, Mendez ML, Rivero A, Garcia J (2000). Amino acid losses during hemodialysis with polyacrylonitrile membranes: effect of intradialytic amino acid supplementation on plasma amino acid concentrations and nutritional variables in nondiabetic patients. Am J Clin Nutr.

[26] VanDorsten JP, Hulsey TC, Newman RB, Menard MK (1998). Fetal anomaly detection by second-trimester ultrasonography in a tertiary center. Am J Obstet Gynecol.

